# Combination of Sequential Organ Failure Assessment (SOFA) score and Charlson Comorbidity Index (CCI) could predict the severity and prognosis of candidemia more accurately than the Acute Physiology, Age, Chronic Health Evaluation II (APACHE II) score

**DOI:** 10.1186/s12879-020-05719-8

**Published:** 2021-01-15

**Authors:** Nobuhiro Asai, Wataru Ohashi, Daisuke Sakanashi, Hiroyuki Suematsu, Hideo Kato, Mao Hagihara, Hiroki Watanabe, Arufumi Shiota, Yusuke Koizumi, Yuka Yamagishi, Hiroshige Mikamo

**Affiliations:** 1 Department of Clinical Infectious Diseases, Aichi Medical University Hospital, 480-1195 1-1 Yazakokarimata, Nagakute, Aichi Japan; 2 Department of Infection Control and Prevention, Aichi Medical University Hospital, Nagakute, Japan; 3 Division of Biostatistics, Clinical Research Center, Aichi Medical University Hospital, Nagakute, Japan

**Keywords:** Candidemia, APACHE II, SOFA score, Bloodstream infections, Prognosis, Sequential organ failure assessment, Charlson comorbidity index, Acute physiology, age, chronic health evaluation II

## Abstract

**Background:**

Candidemia has emerged as an important nosocomial infection, with a mortality rate of 30–50%. It is the fourth most common nosocomial bloodstream infection (BSI) in the United States and the seventh most common nosocomial BSI in Europe and Japan. The aim of this study was to assess the performance of the Sequential Organ Failure Assessment (SOFA) score for determining the severity and prognosis of candidemia.

**Methods:**

We performed a retrospective study of patients admitted to hospital with candidemia between September 2014 and May 2018. The severity of candidemia was evaluated using the SOFA score and the Acute Physiology, Age, Chronic Health Evaluation II (APACHE II) score. Patients’ underlying diseases were assessed by the Charlson Comorbidity Index (CCI).

**Results:**

Of 70 patients enrolled, 41 (59%) were males, and 29 (41%) were females. Their median age was 73 years (range: 36–93 years). The most common infection site was catheter-related bloodstream infection (*n*=36, 51%).The 30-day, and in-hospital mortality rates were 36 and 43%, respectively.

Univariate analysis showed that SOFA score ≥5, APACHE II score ≥13, initial antifungal treatment with echinocandin, albumin < 2.3, C-reactive protein > 6, disturbance of consciousness, and CCI ≥3 were related with 30-day mortality. Of these 7, multivariate analysis showed that the combination of SOFA score ≥5 and CCI ≥3 was the best independent prognostic indicator for 30-day and in-hospital mortality.

**Conclusions:**

The combined SOFA score and CCI was a better predictor of the 30-day mortality and in-hospital mortality than the APACHE II score alone.

**Supplementary Information:**

The online version contains supplementary material available at 10.1186/s12879-020-05719-8.

## Background

Candidemia has emerged as an important nosocomial infection, with a 30–50% mortality rate [1–3]. It is the fourth most common nosocomial bloodstream infection (BSI) in the United States, and the seventh most common nosocomial BSI in Europe and Japan [4–6]. Previously reported risk factors for candidemia include central venous catheterization (CVC), neutropenia, malignancy, abdominal surgery within the previous 30 days, immunosuppressant use and admission to an intensive care unit (ICU). The ubiquity of these risk factors means that most physicians may encounter patients with candidemia. However, there is no established tool or method to evaluate the severity and prognosis of patients with candidemia. The Acute Physiology, Age, Chronic Health Evaluation II (APACHE II) score has been reported to be able to predict the mortality among patients with candidemia [7]. Candidemia patients commonly have severe comorbidity which is responsible for the severity of candidemia. It is reasonable that APACHE II score is useful to predict the prognosis of candidemia as the score includes the evaluation of comorbidity [1–3]. However, in general practice, APACHE II score is very complicated to administer, and its utility in clinical practice is limited. Recently, it has been reported that quick Sequential Organ Failure Assessment (qSOFA) and Sequential Organ Failure Assessment (SOFA) scores are reliable as prognostic tools in the management of sepsis and other infections [8–10]. qSOFA and SOFA consist of 3 to 5 items and are simpler to administer than APACHE II, and are suitable for use by all physicians. Besides, we hypothesize that the Charlson Comorbidity Index (CCI) which is commonly used for the evaluation of comorbidity in general wards, would be appropriate for evaluating the patients’ condition. Therefore, we conducted this retrospective study to assess whether qSOFA and SOFA scores plus CCI could predict mortality in patients with candidemia.

## Methods

### Study design and patient selection

In order to investigate whether SOFA score and the Charlson Comorbidity Index (CCI) could predict the severity and prognosis of patients with candidemia, we performed this retrospective study at the Aichi Medical University hospital, a 900-bed tertiary care center located in Aichi prefecture in central Japan. The study was conducted among patients hospitalized with candidiasis between September 2014 and May 2018. We included patients aged ≥16 years who had hospital-acquired candidemia, which was defined as at least with one positive blood culture of Candida species in patients hospitalized for more than 48 h, with clinical signs and symptoms of infection. Patients who did not have enough information about the disease or those we could not locate because of a transfer to other hospitals were excluded. This study was approved by the Institutional Review Board of Aichi Medical University Hospital (16-H105).

### Severity of candidemia

In medical practice, the severity of the initial presentation of candidemia has been assessed using the APACHE II score [7], qSOFA score, and SOFA score [8–10].

### Definition of variables

Disseminated intravascular coagulation (DIC) was diagnosed according to the diagnostic criteria developed by the Japanese Association for Acute Medicine (JAAM DIC diagnostic criteria) [6]. An altered state of consciousness was defined as Glasgow coma scale (GCS) < 15. Neutropenia was defined as an absolute neutrophil count < 500 × 10^6^/μl.

Antifungal treatment was classified as appropriate or inappropriate depending on whether the identified pathogens were sensitive to the initially prescribed antifungal drugs.

### Performance status

Patients’ general conditions were evaluated by using the Eastern Cooperative Oncology Group (ECOG) performance status (PS) [11] and Karnofsky Performance Status (KPS) [12]. In medical practice, determining PS is an attempt to quantify cancer patients’ general wellbeing and activities of daily living. Recently, this measurement is used to determine whether patients can receive anti-cancer treatment as well as a tool to evaluate conditions such as interstitial lung disease or emphysema [13–15].

### Evaluation of comorbidities

We used the Charlson Comorbidity index (CCI) to evaluate the patients’comorbidities [16, 17]. This index could forecast ten-year mortality for 22 different underlying disease and medical conditions, including cardiac disease, AIDS, and malignancy. Each condition is assigned a score of 1,2,3 or 6 depending on the risk of death, and the sum of these scores is used as the total score to predict mortality.

### Other variables

Clinical data were collected by a review of electronic medical records. Patients’ complete medical records at the time of diagnosis of candidemia were reviewed in our institute. Thirty-five candidate predictors were chosen from published clinical studies as potential prognostic factors [1–6, 18–22]. Continuous variables divided into categories as follows: age (</≥70 years); systemic blood pressure (SBP) (</≥100 mmHg); Glasgow coma scale (GCS) (< 15, 15); white blood cells (WBC) (< 4000, 4000–9000, > 9000 cells/μL); hemoglobin (Hb) (</≥11 g/dL); hematocrit (Ht) (< 30%, 30–46, > 46%); platelets (Plt) (</≥150,000/μL); Sodium (Na) (< 135, 135–140, > 140 mEq/l); Potassium (K) (< 3.5, 3.5–5.0, > 5.0 mEq/L); total bilirubin (TB) (</≥1.2 mg/dL); C-reactive protein (CRP) (</≥6.1 mg/dL); *creatinine* (Cr) (</≥1.2); albumin (Alb) (</≥2.3 g/dL); β-D-glucan (</≥312); APACHE II (</≥13), SOFA score (</≥5), CCI (</≥3). The cut-points for age, CRP, Alb and β-D-glucan were set to the median values, while SBP, GCS, PaO_2_/FiO_2_ ratio, WBC, Hb, Ht, Plt, Na, K, TB and Cr were set at the value that demarcated the normal and abnormal ranges. The cut-points for APACHE II, SOFA score and CCI were set based on the Youden Index [23].

### Identification of candida spp. and susceptibility testing

*Candida* species were identified using the VITEK-MS system (bioMérieux, Marie l’Étoile, France). Susceptibility to amphotericin B, caspofungin, fluconazole, itraconazole, and voriconazole was detected using the AST-YS07 card of VITEK-2 (bioMérieux, Marie l’Étoile, France). The susceptibility of antifungal drugs was assessed with minimum inhibitory concentration (MIC) testing according to the guidelines of the Clinical and Laboratory Standards Institute (CLSI) [24, 25]. MIC values were interpreted according to species-specific clinical breakpoints as established by CLSI for caspofungin (CPFG), fluconazole (FLCZ), itraconazole (ITCZ) (only for *C*. *albicans*), and voriconazole (VRCZ) [24]. Susceptibility to amphotericin B (AMB) and liposomal amphotericin B (L-AMB) were interpreted according to species-specific clinical breakpoints as established by European Committee on Antimicrobial Susceptibility Testing (EUCAST) [25].

### Statistical analyses

The data for categorical variables were reported as percentages, and continuous variables were reported as the mean ± the standard deviation (SD), or the median with the interquartile range (IQR). Chi-square tests or Fisher’s exact tests (two-tailed) were used to compare categorical variables, and unpaired Student’s t-tests or Mann-Whitney U-test were used to compare continuous variables. Logistic regression analysis was used to identify independent risk factors associated with 30-day or in-hospital mortality. Variables with *p*≤0.10 on univariate analyses were entered into the multivariable model. The Hosmer-Lemeshow test was performed to assess the calibration of the model. Receiver-operating characteristic (ROC) curves were evaluated for the predictive values for 30-day or in-hospital mortality. Statistical analyses were performed using SPSS Version 23 for Windows (SPSS Inc*.*, Chicago, IL, USA). A *p*-value< 0.05 was considered statistically significant.

## Results

### Incidence of candidemia

During the study period (941,990 patient-days), the incidence of candidemia was 7.4/100,000 persons. *Candida* spp. was the fourth most common pathogen among patients with bloodstream infections in our institute, after coagulase-negative *Staphylococcus*, *Escherichia coli, and Staphylococcus aureus,* accounting for 7% of positive blood cultures.

### Demographic data

The demographic data and clinical characteristics of the patients, clinical outcomes, pathogens isolated were summarized in Table 1. A total of 70 patients with candidemia were enrolled in this study, of whom 41 (59%) were males and 29 (41%) were females. Their median age was 73 years (range 36–93 years).

**Table 1:** Comparison of candidemia patients among the survival and death group

Variables	All patients (*n*=70)	Death group (*n*=25)	Survival group (*n*=45)	*p*-value
Mean age (years±SD)	72.4±12.1	73.2±13.5	71.9±11.4	0.657
Median (range, years)	73 (36–93)			
Male gender (%)	40 (57)	11 (44)	29 (64)	0.132
Outcome (%)				
30-days mortality	25 (36)			–
In-hospital mortality	30 (43)			–
Length of stay				
Median days (range)	67 (10–418)	56 (11–205)	72 (10–418)	–
Mean days (±SD)	83.1±72.4	64.7±47.5	93.3±82.5	0.106
Performance status				
ECOG-PS (mean±SD)	3.3±0.8	3.8±0.9	3.0±0.4	< 0.001
0–1 (%)	2 (3)	0	2 (4)	
2	11 (16)	0	11 (24)	< 0.001
3	20 (29)	4 (16)	16 (36)	(3,4 v.s.0–2)
4	37 (52)	21 (84)	16 (36)	
KPS (mean±SD)	42.6±17.1	30.3±13.7	49.3±14.7	< 0.001
80–100 (%)	2 (3)	0	2 (4)	
60–70	11 (16)	0	11 (24)	< 0.001
40–50	38 (54)	12 (48)	26 (59)	(< 50 v.s.≧50)
20–30	15 (21)	9 (36)	6 (13)	
≦10	4 (6)	4 (16)	0	
Site of infection (%)				
CRBSI	36 (52)	11 (44)	25 (56)	
Others	5 (7)	3 (12)	2 (4)	0.169
Unknown	29 (41)	12 (48)	17 (38)	
Condition				
Mental altered	33 (47)	18 (72)	15 (33)	0.003
Shock	15 (21)	7 (28)	8 (18)	0.134
DIC	8 (11)	3 (12)	5 (11)	1.000
Endophthalmitis	8/51 (16)	0/10 (0)	8/41 (20)	0.19
Comorbidity (%)				
Chronic heart failure	9 (13)	9 (36)	0	< 0.001
Diabetes mellitus	19 (27)	7 (28)	12 (27)	1.000
Kidney diseases	13 (19)	7 (28)	6 (13)	0.199
Hemodialysis	6 (9)	3 (12)	3 (7)	0.659
Hepatic diseases	5 (7)	2 (8)	3 (7)	1.000
Malignancy	38 (54)	15 (60)	23 (51)	0.617
Chronic respiratory disease	6 (9)	3 (12)	3 (7)	0.659
Gastroesophageal reflex disease	3 (4)	1 (4)	2 (4)	1.000
Dementia	4 (6)	3 (12)	1 (2)	0.127
Cerebrovascular diseases	13 (19)	4 (16)	9 (20)	0.759
Paralysis	2 (3)	1 (4)	1 (2)	1.000
Collagen vascular disease	6 (9)	4 (16)	2 (4)	0.177
Duration until treatment from positive blood culture within 3 days (%)	13 (19)	6 (24)	7 (16)	0.523
External device				
CVC /CV port	53 (76)	20 (80)	33 (73)	0.577
Initial antifungal treatment (%)				
Echinocandins	44 (63)	11 (44)	33 (73)	0.021
L-AMB	18 (26)	8 (32)	10 (22)	0.403
Azole	3 (4)	2 (8)	1 (2)	0.289
Others	5 (7)	4 (16)	1 (2)	0.051
Inappropriate initial therapy	4 (6)	3 (12)	1 (2)	0.127
Evaluation of severity				
APACHE II (mean±SD)	12.9±4.7	15.3±4.1	11.6±4.5	0.001
APACHE II ≧13 (n,%)	40	20 (80)	20 (44)	0.005
SOFA score (mean±SD)	3.9±3.2	5.7±3.1	2.9±2.9	< 0.001
SOFA score ≧5 (n,%)	27 (39)	18 (72)	9 (20)	< 0.001
Charlson Comorbidity Index (mean±SD)	3.5±2.7	4.6±2.4	3.0±2.7	0.012
Charlson Comorbidity Index≧3 (n, %)	41 (59)	21 (84)	20 (44)	< 0.001
Detection of *candida* spp. (n)				
*C. albicans*	27	12	15	0.306
*C. parapsilosis*	20	6	14	0.591
*C. glabrata*	14	3	11	0.35
*C. tropicalis*	7	3	4	0.694
*C. guillermondi*	5	1	4	0.648
*C. dubliniensis*	1	1	0	0.357
Detection of *C. albicans* (v.s. non-*C. albicans*)		12 (48)	15 (33)	0.306
Laboratory data (Mean±SD)				
White blood cell counts (/μl)	10,578.1±6802	12,838.8±7898	9322.2±5833	0.037
Hemoglobin (g/dl)	9.6±1.7	9.6±1.8	9.6±1.7	0.896
Hematocrit (%)	29.0±5.2	28.8±5.4	29.1±5.2	0.823
Platelet counts (× 10^4^/μl)	19.5±11.3	17.7±11.8	20.5±11.1	0.338
Total bilirubin (mg/dl)	1.4±1.8	17.7±11.8	20.5±11.1	0.338
Blood urea nitrogen (mg/dl)	19.5±11.3	1.6±1.7	1.3±1.8	0.514
Creatinine (mg/dl)	1.4±1.7	1.6±1.7	1.3±1.8	0.514
Sodium (mEq/l)	135.5±7.4	133.5±6.6	136.7±7.6	0.082
Potassium (mEq/l)	4.1±0.6	4.0±0.6	4.0±0.6	0.363
Albumin (g/dL)	2.4±0.6	2.2±0.4	2.4±0.6	0.059
C-reactive protein (mg/dL)	7.3±5.2	10.1±5.8	5.7±3.9	< 0.001
β-D-glucan (pg/mL)	847.1±1179.9	831.6±1310.5	855.8±1121.8	0.997

*Two patients had multi-pathogens in blood culture

*CRBSI Catheter related blood stream infection, *CVC* Central venous catheter, *DIC* Disseminated intravascular coagulation, *ECOG* Eastern Cooperative Oncology Group, *KPS* Karnofsy performance status, *L-AMB* Liposomal amphotericin B, *PS* Performance status, *SD* Standard deviation, *SOFA* Sequential Organ Failure Assessment

The patients had a 30-day mortality of 36%, and an in-hospital mortality of 43%. Six of the 10 patients who received an antifungal treatment based on a positive blood culture, died. Of the 68 patients, 27 (40%) received antifungal treatment within 24 h from the onset. The difference in the time to starting antifungal treatment did not differ significantly between those who survived, and those who died (45.6 h vs 36 h, *p*=0.4).

### Microbiological data and antifungal drug selection

#### Detection of Candida spp.

*C*. *albicans* was the most common of the *Candida* species identified, accounting for 39% of the cases, followed by *C*. *parapsilosis* (28%), *C*. *glabrata* (20%), *C*. *tropicalis* (10%) and others (9%). The 30-day mortality according to species was highest in patients with Patients with *C. albicans* candidemia had the highest mortality (44%). *C. albicans* candidemia (vs. non-*C. albicans*) was not associated with an increased 30-day mortality (12/25 [48%] vs. 15/47 [32%], *p*=0.31), but was associated with significantly higher in-hospital mortality (18/33 [55%] vs. 9/37 [24%], *p*=0.01).

The most frequently used initial antifungal treatments were echinocandin (63%), and L-AMB (26%). All candida isolates were susceptible to initial antifungal agents based on CLSI and EUCAST breakpoints. Of the 70 patients, 74% received antifungal treatment within 3 days of the onset, which reflects the period taken for fungus to grow from the blood cultures., and only three patients (4%) did not receive antifungal treatment.

### Relationship between qSOFA, SOFA, APACHE II eastern cooperative oncology group performance status and Karnofsky performance status

The SOFA, APACHE II score and ECOG-PS were higher patients with qSOFA ≥2 than in those with qSOFA < 2. KPS was lower in patients with qSOFA ≥2 than in those with qSOFA< 2 (Table S1).

### Associations between qSOFA, SOFA score and 30-day mortality

**Fig. 1:**
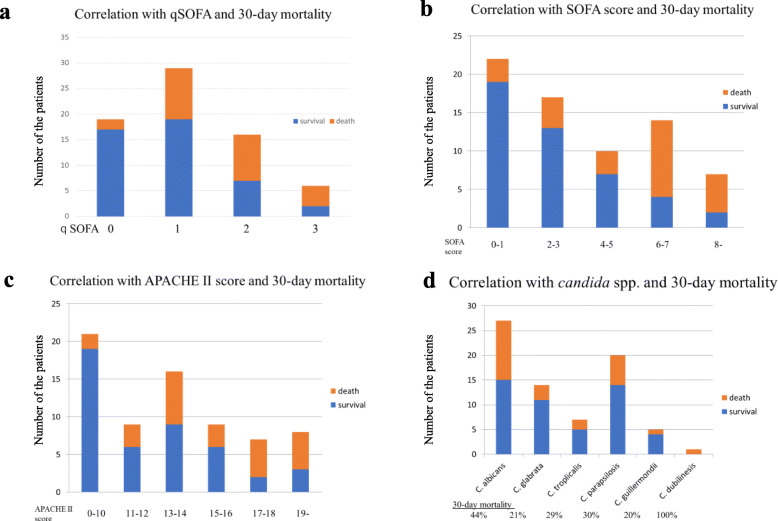
Frequency distributions of: **a** qSOFA score; **b** SOFA score; **c** APACHE II score; and **d** *Candida* species

The associations between qSOFA, SOFA, APACHE II score, *Candida* spp. and 30-day mortality among are shown in Fig. 1. Higher qSOFA and SOFA scores were associated with a higher 30-day mortality. The 30-day mortality of *Candida albicans* was the highest among these patients.

### Relationship between removal of central venous catheters and outcomes

External devices such as CVCs or CV ports were removed in 43 of the 53 (81%) patients. CVCs or CV ports were removed within 24 h from the onset in 29 of the 43 patients (67%). In 37 of the 43 patients (86%), CVCs or CV ports were removed within 3 days of the onset of candidemia. Seven of the 53 patients (13%) did not have their CVCs or CV ports removed. Not removing CVCs or CV ports was a poor prognostic factor (OR 12.5, 95% CI: 1.5–107.6, *p*=0.01). However, removing CVCs or CV ports within 24 h of the onset did not have an observable effect on mortality (OR: 0.8, 95% CI: 0.3–2.3, *p*=0.79).

In terms of the patients with cancer, there were no differences of ECOG-PS and KPS between the patients with and without CVCs or CV ports (data not shown). As for the 28 cancer patients with CVCs or CV ports, not removing CVCs or CV port was a poor prognostic factor (OR 7.4, 95% CI: 1.5–152.3, *p*=0.008). Removing CVCs or CV ports within 24 h of the onset was not a poor prognostic factor (OR 0.6, 95% CI: 0.1–2.5, *p*=0.445).

### Receiver-operating characteristic (ROC) curves of predictive values for 30-day or in-hospital mortality (Figs. 2 and 3)

With respect to the diagnostic value of predictive values for 30-day and in-hospital mortality among candidemia patients, the area under the ROC curve for SOFA score, CCI, APACHE II score, combined SOFA score with CCI and combined APACHE II score with CCI were 0.77 (95% CI:0.65–0.89, *p*< 0.001) and 0.88 (95% CI:0.798–0.962, *p*< 0.001), 0.697 (95%CI:0.567–0.827, *p=*0.007) and 0.753 (95%CI:0.634–0.872, *p*< 0.001), 0.735 (95% CI:0.618–0.852, *p*=0.001) and 0.831 (95% CI:0.738–0.924, *p*< 0.001), 0.79 (95% CI:0.685–0.895, *p*< 0.001) and 0.885 (95% CI:0.809–0.96, *p*< 0.001) and 0.757 (95%CI: 0.643–0.871, *p*< 0.001) and 0.834 (95% CI: 0.742–0.926, *p*< 0.001), respectively.

**Fig. 2:**
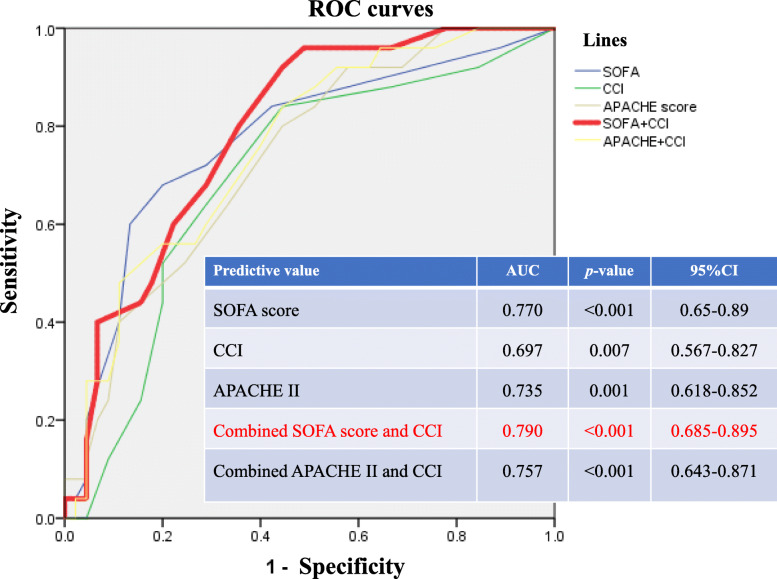
ROC curves of SOFA score, CCI, APACHE II, combined APACHE II and CCI and combined SOFA score and CCI for 30-day mortality

**Fig. 3:**
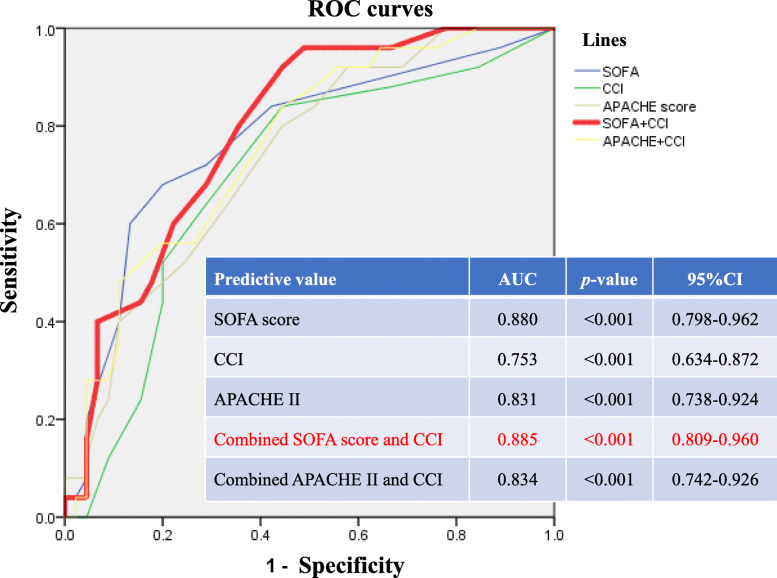
ROC curves of SOFA score, CCI, APACHE II, combined APACHE II and CCI and combined SOFA score and CCI for in-hospital mortality

### Prognostic accuracy of SOFA and APACHE II score for 30-day mortality

Table 2 shows the prognostic accuracy of SOFA and APACHE II for predicting 30-day mortality. The cut-point for the SOFA score was 5. and was selected based on the Youden Index.

**Table 2:** Prognostic accuracy of SOFA score and APACHE II among candidemia patients for 30-day mortality.

Predictive value	Sensitivity (%)	Specificity (%)	PPV (%)	NPV (%)	YI
SOFA score ≧5	68	80	65	82	0.48
SOFA score ≧6	60	87	71	80	0.47
APACHE II ≧13	80	56	50	83	0.36
APACHE II ≧15	52	76	54	74	0.28

*SOFA Sequential Organ Failure Assessment, *YI* Youden index

### Analysis of prognostic factors

The prognostic factors for 30-day mortality are shown in Table 3. Seven of 35 parameters (Table S2) were found to be associated with 30-day mortality in the univariate analysis. These were: SOFA score ≥5, APACHE II score ≥13, initial antifungal treatment with echinocandin, albumin < 2.3, C-reactive protein > 6, disturbance of consciousness, and CCI ≥3. The Hosmer Lemeshow statistic suggested a good fit (χ^2^=11.4, *p*=0.184) in the cohort study.

**Table 3:** Univariate and multivariate analyses among candidemia patients for 30-day death

Variables	Univariate analysis			Multivariate analysis		
	OR	95%CI	*p*-value	OR	95%CI	*p*-value
SOFA score≧5	10.3	3.3–32.1	< 0.001	6.5	1.6–26.4	0.008
APACHE II≧13	5.0	1.6–15.7	0.008			
Echinocandin use as initial treatment	0.3	0.1–0.8	0.021			
Alb < 2.3 g/dl	2.6	0.9–7.0	0.08			
CRP≧6.1 mg/dl	2.7	1.0–7.3	0.081			
Disturbance of consciousness	5.1	1.8–15.0	0.03			
CCI≧3	6.6	1.9–22.2	0.002	4.9	1.1–23.2	0.043

*Alb Albumin, *CCI* the Charlson Comorbidity Index, *CI* Confidential interval, *CRP* C-reactive protein

Of these seven parameters, logistic regression analysis showed that the combination of SOFA score ≥5 and CCI ≥3 were independent prognostic factors for 30-day mortality and in-hospital mortality (Table 4).

**Table 4:** Univariate and multivariate analyses among candidemia patients for in-hospital death

Variables	Univariate analysis			Multivariate analysis		
	OR	95%CI	*p*-value	OR	95%CI	*p*-value
SOFA score≧5	19.0	5.3–68.0	< 0.001	26.4	3.4–202.4	0.002
APACHE II≧13	8.3	2.7–25.3	< 0.001			
Echinocandin use as initial treatment	0.4	0.2–1.1	0.084			
Alb < 2.3 g/dl	4.8	1.7–13.4	0.08			
Disturbance of consciousness	8.3	2.8–24.3	< 0.001			
CCI≧3	10.3	3.2–33.2	< 0.001	15.6	2.1–116.0	0.007

*Alb Albumin, *CCI* the Charlson Comorbidity Index, *CI* Confidential interval, *CRP* C-reactive protein

**CRP is not a poor prognostic factor among candidemia patients for in-hospital death

## Discussion

In contrast to the complicated APACHE II scoring system, which consists of ASP points, age and immunocompromised state, both the SOFA score and CCI are simple to calculate. SOFA is a tool for evaluating the severity of failure of organs such as the kidney or the liver [26]. In addition, while platelet count is included in SOFA score, it is not included in APACHE II. This difference might contribute to the more precise evaluation of patients’ conditions by SOFA compared to APACHE II in seriously ill patients with conditions such as disseminated intravascular coagulation or multiple organ failure. In this study, 23 of the 70 patients were diagnosed as having candidemia in ICU, while the remaining 47 patients were diagnosed in a general ward. Table S1 showed that the patients with qSOFA≧2 could reflect the disease severity of SOFA score and APACHE II score. In a general ward, patients’ condition should be evaluated by qSOFA [27] (γ=0.505 with Spearman’s rank correlation test). The results showed the validity of qSOFA among candidemia patients in a general ward. The severity and prognosis of candidemia could be evaluated by SOFA score more reliably than by APACHE II. CCI is a useful tool for evaluating comorbid conditions in patients with underlying diseases [13, 14]. Furthermore, the outcome of candidemia can be affected by patients’ general condition and underlying diseases such as malignancy. Thus, it is reasonable that combined SOFA score and CCI could more precisely predict the severity and prognosis of patients with candidemia.

Removal of CVCs is considered to be a standard procedure among patients with candidemia [27, 28]. Although it has been reported that removing CVCs within 24 h of the onset of candidemia is associated with a reduced mortality rate [29], our study results did not confirm this finding. These discrepancies may be attributable to the lack of uniformity of variables and differences in both previous studies and ours. We found that patients whose CVCs were removed, showed better PSs and had lower SOFA scores than those whose CVCs were not removed (Table 5).

**Table 5:** Comparison between score values of candidemia patients who removed or did not remove CVCs

Score values (Mean±SD)	Removal of CVCs (*n*=43)	Non-removal of CVCs (*n*=10)	*p*-value
ECOG-PS	3.1±0.9	3.8±0.4	0.019**9**
KPS	49.1±16.	27±16.4	< 0.001
SOFA score	3.4±3.4	6.3±2.6	0.021
APACHE II	11.5±4.9	16.1±4.4	0.008
CCI	3.2±2.8	5.6±2.3	0.014

*CCI the Charlson Comorbidity Index, *CVC* Central venous catheter, *ECOG* Eastern Cooperative Oncology Group, *KPS* Karnofsy performance status, *PS* Performance status, *SD* Standard deviation, *SOFA* Sequential Organ Failure Assessment

We can assume that physicians tended to remove CVCs of patients in good general condition, whose life expectancy is considered favorable. Conversely, physicians might be hesitant to remove CVCs of patients with poor general condition.

Candidemia is almost always primarily of gastrointestinal origin in patients with cancer who have severe neutropenia and mucositis, and removal of CVCs is less likely to have an impact on the outcome in this setting. However, we believe that removal of CVCs is appropriate in the treatment of patients with candidemia considering that CVCs are foreign bodies.

On insertion of CVCs, physicians should take into account the ease and safety of CVC removal when candidemia is suspected, and select catheter device. Peripherally inserted central venous catheters could be removed easier and safer than CV port.

This study has several limitations. Firstly, it is a retrospective study with a relatively low sample size. Thus, there might be a bias in the data selection and analysis. Secondly, we enrolled only patients with candidemia diagnosed by a blood culture. Generally, 50% of individuals with candidemia have negative blood cultures. Thus, the patients in our study may not have been representative of all individuals with candidemia.

## Conclusions

Combined SOFA score and CCI could possibly be a more accurate predictor of severity and prognosis among patients with candidemia than the APACHE II score for 30-day, or in-hospital death.

## Supplementary Information

**Additional file 1: Table S1.** Comparison with predictive values among candidemia patients with qSOFA≧2 and those with < 2.

**Additional file 2: Table S2.** Prognostic factor for 30-day mortality by an univariate analysis.

## Data Availability

All data generated or analyzed during this study are included in this published article and are found in result session and supplementary information (Additional files 1 and 2).

## Abbreviations

AMB: Amphotericin B
APACHE II: Acute Physiology, Age, Chronic Health Evaluation II
AUROC: Area under the receiver-operating characteristic curve
Alb: Albumin
BSI: Blood stream infection
CCI: Charlson Comorbidity Index
CLSI: Clinical and Laboratory Standards Institute
CPFG: Caspofungin
CRP: C-reactive protein
CVC: Central venous catheter
Cr: *Creatinine*
DIC: Disseminated intravascular coagulation
ECOG: Eastern Cooperative Oncology Group
EUCAST: European Committee on Antimicrobial Susceptibility Testing
FLCZ: Fluconazole
GCS: Glasgow coma scale
Hb: Hemoglobin
Ht: Hematocrit
ICU: Intensive care unit
IQR: Interquartile range
ITCZ: Itraconazole
JAAM: Japanese Association for Acute Medicine
K: Potassium
KPS: Karnofsky Performance Status
L-AMB: Liposomal amphotericin B
MIC: Minimum inhibitory concentration
Na: Sodium
PS: Performance status
Plt: Platelets
SBP: Systemic blood pressure
SD: Standard deviation
SOFA: Sequential Organ Failure Assessment
TB: Total bilirubin
VRCZ: Voriconazole
WBC: White blood cells
qSOFA: Quick Sequential Organ Failure Assessment
