# Supplementary material for: Combination of Sequential Organ Failure Assessment (SOFA) score and Charlson Comorbidity Index (CCI) could predict the severity and prognosis of candidemia more accurately than the Acute Physiology, Age, Chronic Health Evaluation II (APACHE II) score
Source: BMC Infect Dis. 2021 Jan 15;21:77. doi: 10.1186/s12879-020-05719-8 (PMC7811217; doi:10.1186/s12879-020-05719-8)
Supplement: Supplementary file 1 — Additional file 1: Table S1. Comparison with predictive values among candidemia patients with qSOFA≧2 and those with < 2. [file 12879_2020_5719_MOESM1_ESM.docx]

Table S1. Comparison with predictive values among candidemia patients with qSOFA≧2 and those with <2

| Predictive value  (Mean±SD) | qSOFA≧2 | qSOFA<2 | *p*-value |
| --- | --- | --- | --- |
| SOFA score | 5.8±3.1 | 2.8±2.8 | <0.001 |
| APACHE II score | 15.7±4.7 | 11.4±4.0 | <0.001 |
| CCI | 4.2±2.9 | 3.2±2.5 | 0.104 |
| ECOG-PS | 3.9±0.3 | 3.0±0.9 | <0.001 |
| KPS | 32.4±11.6 | 48.2±17.1 | <0.001 |
