# Supplementary material for: Combination of Sequential Organ Failure Assessment (SOFA) score and Charlson Comorbidity Index (CCI) could predict the severity and prognosis of candidemia more accurately than the Acute Physiology, Age, Chronic Health Evaluation II (APACHE II) score
Source: BMC Infect Dis. 2021 Jan 15;21:77. doi: 10.1186/s12879-020-05719-8 (PMC7811217; doi:10.1186/s12879-020-05719-8)
Supplement: Supplementary file 2 — Additional file 2: Table S2. Prognostic factor for 30-day mortality by an univariate analysis. [file 12879_2020_5719_MOESM2_ESM.doc]

Table S2. Prognostic factor for 30-day mortality by an univariate analysis [1-6, 16-20]

| Variables | Odds ratio | 95%CI | *p*-value |
| --- | --- | --- | --- |
| Age (≧70 v.s. <70 years) | 0.8 | 0.3-2.1 | 0.611 |
| Sex (male v.s. female) | 0.4 | 0.2-1.2 | 0.132 |
| Mental altered (GCS<15) | 5.1 | 1.8-15.0 | 0.003 |
| Systemic blood pressure<100mmHg  or vasopressor use | 2.5 | 0.9-7.1 | 0.114 |
| Disseminated intravascular coagulation | 2.6 | 0.6-10.6 | 0.265 |
| Chronic heart failure | 24.8 | 2.9-211.2 | <0.001 |
| Diabetes mellitus | 1.1 | 0.4-3.2 | 1.000 |
| Kidney diseases | 2.5 | 0.7-8.6 | 0.199 |
| Hemodialysis | 1.9 | 0.4-10.3 | 0.659 |
| Hepatic diseases | 1.2 | 0.2-7.8 | 1.000 |
| Malignancy | 1.4 | 0.5-3.9 | 0.617 |
| Chronic respiratory disease | 1.9 | 0.4-10.3 | 0.659 |
| Gastroesophageal reflex disease | 0.9 | 0.1-10.4 | 1.000 |
| Dementia | 6.0 | 0.6-61.1 | 0.127 |
| Cerebrovascular diseases | 0.8 | 0.2-2.8 | 0.759 |
| Paralysis | 1.8 | 0.1-30.6 | 1.000 |
| Collagen vascular disease | 4.1 | 0.7-24.2 | 0.177 |
| Duration until treatment from positive blood culture within 3 days | 1.7 | 0.5-5.8 | 0.523 |
| Echinocandins as Initial  antifungal treatment | 0.3 | 0.1-0.8 | 0.021 |
| Inappropriate anti-fungal therapy as initial treatment | 6.0 | 0.6-61.1 | 0.127 |
| APACHE II≧13 | 5.0 | 1.6-15.7 | 0.008 |
| SOFA score≧5 | 10.3 | 3.3-32.1 | <0.001 |
| Charlson Comorbidity Index≧3 | 6.6 | 1.9-22.2 | 0.002 |
| Detection of *C.albicans*  (v.s. non-C.albicans) | 1.9 | 0.7-5.0 | 0.306 |
| White blood cell counts (/μl) <4000, ≧9000 | 4.6 | 1.4-15.6 | 0.018 |
| Hemoglobin (g/dl) <11 | 0.7 | 0.2-2.3 | 0.772 |
| Hematocrit (%) <30 | 1.6 | 0.6-4.3 | 0.455 |
| Platelet counts (/μl) <150,000 | 1.7 | 0.6-4.8 | 0.308 |
| Total bilirubin (mg/dl)≧1.2 | 2.2 | 0.7-6.6 | 0.169 |
| Creatinine (mg/dl)≧1.2 | 1.6 | 0.6-4.6 | 0.428 |
| Sodium (mEq/l) <135 | 2.3 | 0.8-6.1 | 0.137 |
| Potassium (mEq/l)≧5.1, <3.5 | 1.0 | 0.3-3.4 | 1.000 |
| Albumin (g/dL) <2.3 | 2.6 | 0.9-7.0 | 0.08 |
| C-reactive protein (mg/dL)≧6.1 | 2.7 | 1.0-7.3 | 0.081 |
| β-D-glucan (pg/mL)≧312 | 2.0 | 0.6-6.9 | 0.341 |

＊CI, confidence interval; GCS, Glasgow coma scale; SOFA, Sequential Organ Failure Assessment.
